# Comparison of Glucose and Satiety Hormone Response to Oral Glucose vs. Two Mixed-Nutrient Meals in Rats

**DOI:** 10.3389/fnut.2018.00089

**Published:** 2018-09-26

**Authors:** Danielle T. Vannan, Marc R. Bomhof, Raylene A. Reimer

**Affiliations:** ^1^Faculty of Kinesiology, University of Calgary, Calgary, AB, Canada; ^2^Department of Kinesiology and Physical Education, University of Lethbridge, Lethbridge, AB, Canada; ^3^Department of Biochemistry and Molecular Biology, Cumming School of Medicine, University of Calgary, Calgary, AB, Canada

**Keywords:** glycemia, satiety hormones, oral glucose tolerance test, meal tolerance test, glucose response

## Abstract

The obesity epidemic is driving interest in identifying strategies that enhance appetite control by altering the secretion of hormones that regulate satiety and food intake. An appropriate nutrient stimulus, such as a meal or oral nutrient solution, is needed to elicit the secretion of satiety hormones in order to evaluate the impact of dietary and other interventions. Our objective was to compare the effects of oral glucose vs. mixed nutrients on plasma concentrations of glucose and appetite-regulating hormones to determine the most appropriate oral nutrient challenge to trigger robust hormone secretion. A 120 min oral glucose tolerance test (OGTT) was compared with two meal tolerance tests (MTT) of differing formulation to evaluate glucose and satiety hormone responses. Following overnight feed deprivation, male Sprague-Dawley rats were given one of three oral gavages with equal carbohydrate content (2 g CHO/kg) in the form of: (1) Dextrose, (2) Ensure®, or (3) Mixed Meal. A fourth group was given saline as a control. Blood was collected via tail snip and analyzed for glucose, insulin, GLP-1, GIP, PYY, amylin, leptin, and ghrelin. Dextrose resulted in the highest blood glucose at T15 (*P* = 0.014), while the mixed meal was significantly higher than saline from T30-T120 (*P* < 0.05). Insulin was higher at T15 with dextrose compared to saline (*P* = 0.031) and Ensure® (*P* = 0.033). GLP-1 tAUC was significantly higher with dextrose compared to mixed meal (*P* = 0.04) while GIP tAUC was higher with dextrose and mixed meal compared to saline (*P* < 0.05). Changes in tAUC for insulin, amylin, leptin, ghrelin, and PYY did not reach significance. Based on these findings, dextrose appears to provide a robust acute glycemic and hormone response and is therefore likely an appropriate oral solution to reproducibly test the impact of various dietary, surgical, or pharmacological interventions on glucose and satiety hormone response.

## Introduction

Substantial resources have been dedicated to the treatment of obesity and related comorbidities including diabetes, hypertension, and cardiovascular disease (1). Of current interest are strategies that target appetite control and feeding behavior (2). The hormonal signaling network that allows for cross-talk between the brain and peripheral tissues about energy stores and metabolic status includes leptin, insulin, glucagon-like peptide-1 (GLP-1), peptide YY (PYY), and ghrelin among others (3). Exposure to either an oral glucose tolerance test (OGTT) or a meal tolerance test (MTT) is a common protocol used to demonstrate changes in the release of satiety hormones in response to an acute stimulus such as a drug or nutrient or following longer term interventions to assess the effects of treatments such as bariatric surgery or dietary change on satiety hormones. Indeed, a variety of nutrients and/or dietary patterns have been shown to alter the secretion of orexigenic and anorexigenic hormones in rodents, including work with the prebiotics inulin and oligofructose and whey protein, that in turn are associated with changes in body weight and adiposity (4, 5). Furthermore, studies showing substantial changes in the release of appetite-regulating hormones following bariatric surgery have helped to identify one of several potential mechanisms by which this surgery enhances weight loss and leads to diabetes resolution (6, 7).

Oral gavage of a glucose solution or a complete meal is the most common technique used to assess an animals' satiety hormone response to nutrients (8–10). While individual studies may utilize either an OGTT or MTT to assess effects on satiety hormone secretion, to our knowledge they have not been directly assessed in a head to head manner. This is a critical comparison given that the two techniques, which deliver a single macronutrient in the OGTT and all three macronutrients in the MTT, could elicit distinct responses that could influence the interpretation of outcomes across different studies evaluating dietary or therapeutic interventions. For example, carbohydrate is highly effective at suppressing ghrelin release but fat is a rather weak ghrelin-suppressor (11). Similarly, individual macronutrients vary in their stimulation of GLP-1 release (12). It was our goal in this methods paper to identify the most appropriate test to elicit a robust response in both blood glucose and appetite-regulating hormone concentrations in rats. Since multiple hormones are part of the hormonal signaling network that controls appetite and feeding behavior (3), we measured the circulating levels of glucose, insulin, GLP-1, glucose-dependent insulinotropic peptide (GIP), peptide tyrosine tyrosine (PYY), leptin, amylin, and ghrelin during an OGTT and MTT (13). Based on evidence that all macronutrients stimulate GLP-1 secretion in the gut (14–16), we hypothesized that a MTT would elicit greater overall secretion of GLP-1 due to the mixed macronutrient composition compared to an OGTT. We predicted that other related hormones, such as PYY would be similarly affected.

## Materials and methods

### Animals and housing

This study was carried out in accordance with recommendations of the *Guide to the Care and Use of Experimental Animals*, Canadian Council on Animal Care. The protocol was approved by The University of Calgary Animal Care Committee. Twenty-four male Sprague-Dawley rats (10 weeks of age) were obtained from Charles River (Montreal, QC, Canada) and housed 3 per cage on a 12 h light–dark cycle in a temperature and humidity controlled room. Rats were maintained on standard rat chow (Lab Diet #5001, St. Louis, MO).

### Preparation of oral test solutions

All oral test solutions were created with equal carbohydrate (CHO) concentrations (0.5 g CHO/ml of solution). The 50% dextrose solution (wt/vol) was prepared by dissolving 0.5 g of dextrose in 1 ml of purified water. Commercial Ensure Plus® vanilla flavor [0.21 g carbohydrate(CHO)/ml: 0.086 g/ml sugar and 0.128 g/ml maltodextrin] was used as a mixed meal solution. Because of the lower carbohydrate content of Ensure®, dextrose was added to standardize carbohydrate content (adjusted to 0.5 g CHO/ml by adding in 0.44 g of dextrose/ml). The final macronutrient distribution of the Ensure® test meal was 80% CHO, 13% Fat, and 7% Protein. The in-house mixed meal was prepared by combining 0.5 g/ml dextrose, 0.092 g/ml vegetable oil, 0.149 g/ml high nitrogen casein (87% protein) (Dyets Inc., Bethlehem, PA) in purified water. The macronutrient distribution of the in-house mixed test meal was 60% CHO, 25% Fat, and 15% protein. The energy content for each of the experimental diets was: Dextrose-8.4 KJ/ml; Ensure^®^−10.4 KJ/ml; and Mixed-meal-13.9 KJ/ml. A full description of the nutrient composition of the test diets is provided in Table 1.

**Table 1:** Macronutrient composition and energy content of oral meal test solutions.

	**Oral glucose solution**	Commercial Ensure Plus^®^ vanilla flavor*	**In-house mixed meal solution**
Total CHO, g/ml (KJ/ml)	0.5 (8.4)	0.5 (8.4)	0.5 (8.4)
Source	Dextrose	Corn maltodextrin, sucrose	Dextrose
Percent of macronutrients	100%	80%	60%
Total Protein, g/ml (KJ/ml)	–	0.043 (0.72)	0.125 (2.09)
Source	–	Calcium caseinate, soy protein	High-nitrogen casein
Percent of macronutrients	–	7%	15%
Total Fat, g/ml (KJ/ml)	–	0.035 (1.32)	0.092 (3.47)
Source	–	Canola oil, corn oil	Vegetable oil
Percent of macronutrients	–	13%	25%
Micronutrient content	Absent	^#^As per manufacturer	Negligible
Total KJ/ml	8.4	10.4	13.9

* *Composition derived from information available online from Abbott Nutrition. Note that the macronutrient composition differs slightly from the commercial formulation due to the standardization of carbohydrate (i.e. addition of dextrose) across all solutions as described in the section Materials and Methods*.

# *A detailed breakdown of the micronutrients is available from: https://nutrition.abbott/ca/en/oral-nutritional-supplements*.

### Experimental protocol

One day prior to testing, rats (*n* = 6) were weighed and randomly assigned to 1 of 4 test groups: (1) Dextrose; (2) Ensure®; (3) Mixed meal; or (4) Saline. Following overnight feed deprivation with access to water, rats were given an oral gavage of the test solutions standardized to provide 2 g CHO/kg body weight or an equivalent volume of saline. Blood was sampled via tail nick at 0, 15, 30, 60, 90, and 120 min post-gavage and immediately analyzed for glucose using a blood glucose meter (OneTouch Glucose Meter, Lifescan Inc., Milpitas, CA). At the same time, blood was collected in a chilled tube containing diprotinin-A (0.068 mg/ml blood; MP Biomedicals, Irvine, CA), Sigma protease inhibitor (1 mg/ml blood; Sigma Aldrich, Oakville, ON, Canada) and Roche Pefabloc (1 mg/ml of blood; Roche, Mississauga, ON, Canada). Plasma was stored at −80°C until analysis for satiety hormones.

### Appetite-regulating hormone analysis

Concentrations of acylated ghrelin, active amylin, insulin, leptin, total GIP, active GLP-1, and total PYY were quantified using a commercially-available Rat Gut Hormone Panel Milliplex kit (Millipore, St. Charles, MO) and Luminex instrument according to the manufacturer's specifications. The intra-assay CV is < 10% and the inter-assay CV is < 15% for all analytes.

### Statistics

All data are presented as mean ± standard error of the mean (SEM). Repeated measures ANOVA was used to assess differences in glucose and hormone response using time as the within subject factor and diet as the between subjects factor. If a significant time by diet interaction was identified, Tukey's *post-hoc* test was used to determine wherein the differences were found. For AUC data, a one-way ANOVA followed by Tukey's *post-hoc* test was conducted using SPSS V24.0 software (SPSS Inc., Chicago, IL, USA). Data was considered significant at *P* < 0.05.

## Results

### Body weight

There were no significant differences in body weight between groups (saline 404.5 g ± 6.0, dextrose 404.4 g ± 9.6, Ensure® 404.5 g ± 4.3, mixed meal 405.8 g ± 2.7; *P* = 0.99).

### Glucose and insulin response

**Figure 1:**
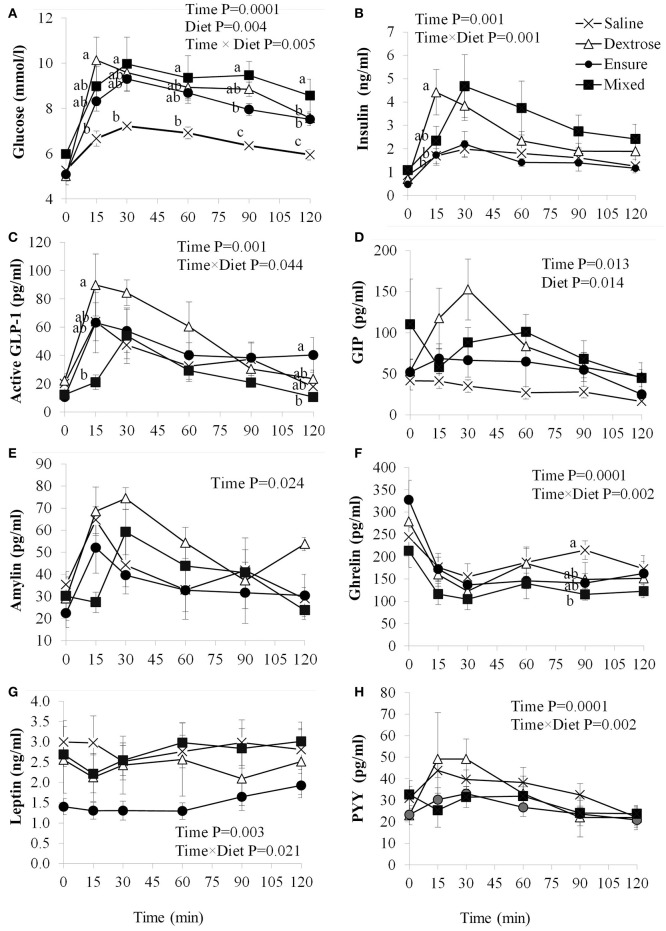
Plasma glucose and satiety hormone response following an oral load of saline, dextrose, Ensure®, or a mixed meal. **(A–H)** Values are mean±SEM (*n* = 6). Differences between the oral solutions were measured using one-way ANOVA at each time point. Values with different superscripts are significantly different (*P* < 0.05).

Blood glucose concentrations were affected by time (*P* = 0.0001), diet (*P* = 0.004) and the interaction of time × diet (*P* = 0.005; Figure 1A). There were no differences in fasting blood glucose levels between groups, however, compared to saline, dextrose elicited the highest blood glucose at T15 (*P* = 0.014) which was not statistically different from the MTT groups. At T30, mixed meal treatment was significantly higher than saline (*P* = 0.046) and continued to be at T60 (*P* = 0.048), T90 (*P* = 0.0001), and T120 (*P* = 0.001). Dextrose and Ensure® also elicited higher blood glucose concentrations than saline at T90 and T120 (*P* < 0.05). Mixed meal blood glucose concentrations were also significantly higher than Ensure® at T90 (*P* = 0.049). Total AUC for glucose was significantly higher following dextrose (*P* = 0.017) and mixed meal (*P* = 0.005) treatments compared to saline (Table 2). Insulin concentrations were significantly influenced by time (*P* = 0.001) and time × diet (*P* = 0.001; Figure 1B). At T15, dextrose elicited higher insulin compared to saline (*P* = 0.031) and Ensure® (*P* = 0.033). There were no differences in total AUC for insulin (Table 2).

**Table 2:** Total AUC for glucose and satiety hormones in rats administered saline, dextrose, Ensure®, or a mixed meal (mean ± SEM).

	**Saline**	**Dextrose**	**Ensure^®^**	**Mixed meal**
	**AUC**	**AUC**	**AUC**	**AUC**
Glucose (mM)	758.8 ± 15.6^a^	1047.1 ± 41.8^b^	979.7 ± 35.7^ab^	1092.4 ± 95.7^b^
Insulin (ng/ml)	196.9 ± 36.4	311.3 ± 45.9	179.8 ± 32.4	378.3 ± 100.0
GLP-1 (pg/ml)	4399.8 ± 768.8^ab^	6079.0 ± 782.5^a^	5236.6 ± 1334.8^ab^	2722.1 ± 338.3^b^
GIP (pg/ml)	3584.6 ± 712.1^a^	10472.7 ± 911.4^b^	6844.9 ± 2231.0^ab^	9378.4 ± 1359.6^b^
Amylin (pg/ml)	3738.4 ± 941.2	5028.0 ± 758.1	2498.5 ± 881.6	3860.5 ± 736.2
Ghrelin (ng/ml)	22.5 ± 2.9	19.4 ± 3.0	19.1 ± 1.8	15.1 ± 2.2
Leptin (ng/ml)	336.5 ± 58.3	281.9 ± 89.1	175.7 ± 28.6	329.0 ± 50.9
PYY(pg/ml)	3965.8 ± 581.9	3446.5 ± 1007.7	3046.2 ± 342.3	3359.1 ± 473.5

*Data are expressed as mean ± SEM (n = 6). Values with different superscripts are significantly different (P < 0.05)*.

### Appetite-regulating hormone response

GLP-1 concentrations were significantly influenced by time (*P* = 0.001) and time × diet (*P* = 0.044; Figure 1C). Dextrose elicited a greater GLP-1 response than mixed meal at T15 (*P* = 0.052) although it was not different from Ensure® and saline. At T120, GLP-1 was higher with Ensure® compared to mixed meal (*p* = 0.042). Total AUC for GLP-1 was significantly higher for dextrose compared to mixed meal (*P* = 0.04; Table 2). Plasma GIP was affected by time (*P* = 0.013) and diet (*P* = 0.014; Figure 1D). Dextrose (*P* = 0.014) and mixed meal (*P* = 0.043) elicited higher total AUC for GIP compared to saline (Table 2). Plasma amylin was only influenced by time (*P* = 0.024; Figure 1E). There were no differences in amylin total AUC (Table 2). Plasma ghrelin was significantly influenced by time (*P* = 0.0001) and time × diet (*P* = 0.001; Figure 1F) with saline having significantly higher ghrelin concentrations compared to mixed meal at T90 (*P* = 0.049. There were no differences in total AUC, however, for ghrelin (Table 2). Both leptin (Figure 1G) and PYY (Figure 1H) were significantly influenced by time (*P* < 0.003) and time × diet (*P* < 0.021), however, *post-hoc* analysis did not show any significant differences across the time points. There were no differences among groups for total AUC for leptin and PYY (Table 2).

## Discussion

Altering circulating satiety hormone levels has emerged as an important target in the treatment and prevention of obesity and associated metabolic disease. The continued development of novel neurohormonal therapeutic strategies for obesity highlights the critical need for preclinical studies to be able to elicit a robust and measurable satiety hormone response (17). Therefore, this experiment evaluated glycemic response and appetite-regulating hormone concentrations across various acute oral nutrient loads. It was anticipated that the secretion of one of the primary satiety hormones of interest, GLP-1, would be greatest following the mixed meal due to the combined macronutrient composition of the treatment (13) and that other related hormones, such as the other incretin GIP or PYY which is co-expressed with GLP-1 in intestinal L cells (18), would react similarly. We also chose to compare a mixed meal treatment that was prepared in-house with the commercially available liquid meal replacement, Ensure®.

We found that both the dextrose and mixed meal treatments elicited a greater blood glucose response compared to saline. This pattern was also reflected in the total AUC for GIP. However, for GLP-1 the dextrose challenge resulted in higher concentrations compared to the mixed meal treatment. This observation may relate to the incretin properties of GLP-1 in which the more rapid rise in blood glucose from baseline to T15 following dextrose treatment elicited a greater response than the slower blood glucose rise seen with the mixed meal treatment (19). The pattern of insulin release over the 120 min was similar to glucose, thereby suggesting a consistent pattern of glycemic and insulinemic response. Amylin is co-secreted with insulin and the changes in amylin from baseline to T15 also reflected insulin secretion with dextrose but less so with the mixed meal (20).

The changes observed between the treatment groups for the orexigenic hormone ghrelin were similar to what we expected. Saline reached a significantly higher concentration at T90 compared to the mixed meal, reflecting a pattern of ghrelin secretion that is typically highest before a meal and subsequently decreases following food consumption (21). Despite finding a significant time × diet effect for leptin and PYY, *post-hoc* analysis suggested that the four nutrient loads had minimal effect on plasma leptin and PYY concentrations.

An interesting observation in the study was that Ensure®, relative to the other carbohydrate containing solutions, did not elicit a robust appetite-regulating hormone response. Compared to saline, Ensure® was not associated with significantly higher tAUC for any of the outcome variables examined and only one time point (T120) for one hormone (GLP-1) was significantly higher with Ensure® vs. mixed meal. The reason for the blunted response with Ensure® is not clear, but one possibility is that Ensure® contains maltodextrin as a portion of the total carbohydrate content, whereas the carbohydrate in the other solutions was pure dextrose. Maltodextrins are classified according to dextrose equivalents (DE) ranging from 3 to 20 and depending on the DE used in the commercial product, could affect glycemia (22). Furthermore, Ensure® is a commercial meal replacement beverage and contains a full complement of micronutrients. The combined effect of these two notable differences in the composition of Ensure® could explain the blunted glucose and satiety hormone response. These findings are potentially of interest since some scientists may use a commercial meal replacement solution to examine satiety hormone response and the blunted response may mask the true effect of the experimental intervention being tested. While we chose to standardize our formulations based on carbohydrate delivery to the animals, this is a limitation because we did not equalize other components such as the micronutrients or the energy content. Future studies that control for other factors such as this would be needed to determine the influence of these nutrients and compositional factors on satiety hormone release.

While dextrose and mixed meal treatments produced largely similar appetite-regulating hormone profiles, the notable difference was the heightened response of GLP-1 to the dextrose treatment. Given that changes in GLP-1 secretion in response to nutrients and other interventions is of interest to the fields of obesity and diabetes, the robust glycemic and satiety hormone response produced by dextrose and its ease of formulation, may make it an appropriate, if not superior, means of identifying the effects of experimental diets/therapies on biochemical markers of appetite. Differences in the response of some satiety hormones to the two mixed meal formulations suggest that dextrose may represent a more reproducible nutrient stimulus across multiple studies.

## Author contributions

DV and MB were responsible for data acquisition, data analysis and drafting the manuscript. RR was responsible for the study conception and design, data analysis and interpretation. All authors critically reviewed the work and approved the final version.

### Conflict of interest statement

The authors declare that the research was conducted in the absence of any commercial or financial relationships that could be construed as a potential conflict of interest. The reviewer IS and handling Editor declared their shared affiliation.
